# Hedgehog Pathway Inhibitors as Targeted Cancer Therapy and Strategies to Overcome Drug Resistance

**DOI:** 10.3390/ijms23031733

**Published:** 2022-02-03

**Authors:** Ngoc Minh Nguyen, Jungsook Cho

**Affiliations:** College of Pharmacy and Integrated Research Institute for Drug Development, Dongguk University-Seoul, Goyang 10326, Gyeonggi, Korea; nguyenngocminh.blue@gmail.com

**Keywords:** hedgehog signaling, hedgehog inhibitor, smoothened inhibitor, basal cell carcinoma, drug resistance, targeted cancer therapy

## Abstract

Hedgehog (Hh) signaling is a highly conserved pathway that plays a vital role during embryonic development. Recently, uncontrolled activation of this pathway has been demonstrated in various types of cancer. Therefore, Hh pathway inhibitors have emerged as an important class of anti-cancer agents. Unfortunately, however, their reputation has been tarnished by the emergence of resistance during therapy, necessitating clarification of mechanisms underlying the drug resistance. In this review, we briefly overview canonical and non-canonical Hh pathways and their inhibitors as targeted cancer therapy. In addition, we summarize the mechanisms of resistance to Smoothened (SMO) inhibitors, including point mutations of the drug binding pocket or downstream molecules of SMO, and non-canonical mechanisms to reinforce Hh pathway output. A distinct mechanism involving loss of primary cilia is also described to maintain GLI activity in resistant tumors. Finally, we address the main strategies to circumvent the drug resistance. These strategies include the development of novel and potent inhibitors targeting different components of the canonical Hh pathway or signaling molecules of the non-canonical pathway. Further studies are necessary to avoid emerging resistance to Hh inhibitors and establish an optimal customized regimen with improved therapeutic efficacy to treat various types of cancer, including basal cell carcinoma.

## 1. Introduction

The Hedgehog (Hh) signaling pathway plays crucial roles in embryonic development, transmitting information to regulate cell growth and differentiation [1,2,3]. It is a highly conserved evolutionary pathway, almost silent in the adult except for a few tissues such as the skin [4,5]. Specifically, the Hh pathway is a notable example of extracellular morphogenic signal, mitogen, cell survival factor, and axon guidance factor [6,7,8,9]. Recently, the Hh signaling pathway has been recognized as one of the most intensely investigated targets for cancer treatment. Aberrant activation of this pathway was found in various types of cancer, such as basal cell carcinoma (BCC), medulloblastoma, breast cancer, lung cancer, etc. [10,11,12,13,14]. The first group of inhibitors targeting the Hh pathway are Smoothened (SMO) antagonists, with the first approval for BCC treatment [15,16]. However, as in other targeted cancer therapies, drug resistance arose and diminished the efficacy of these inhibitors. In the present review, we briefly overviewed canonical and non-canonical Hh pathways and their roles in the normal context as well as in cancer. In addition, we summarized the development of SMO inhibitors as targeted cancer therapy. Finally, we addressed the mechanisms underlying the resistance of tumors to SMO inhibitors and the main strategies that could be applied to overcome the drug resistance.

## 2. Overview of the Hh Signaling Pathway

### 2.1. Canonical Hh Signaling Pathway

The Hh signaling pathway is dependent on a highly specialized organelle, the primary cilium, to regulate tissue patterning and homeostasis [17]. The primary cilium is a small cellular projection found on most vertebrate cells that acts as a cellular antenna, where membrane receptors and signaling components are concentrated. Whereas invertebrates have only one Hh ligand, vertebrates have three—sonic hedgehog (Shh), desert hedgehog (Dhh), and Indian hedgehog (Ihh). Ihh and Dhh expression patterns appear to be somewhat tissue-specific, while Shh is expressed in diverse organs both in early development and in adults, including central nervous system, limbs, and many other parts of the body [18,19,20,21].

**Figure 1:**
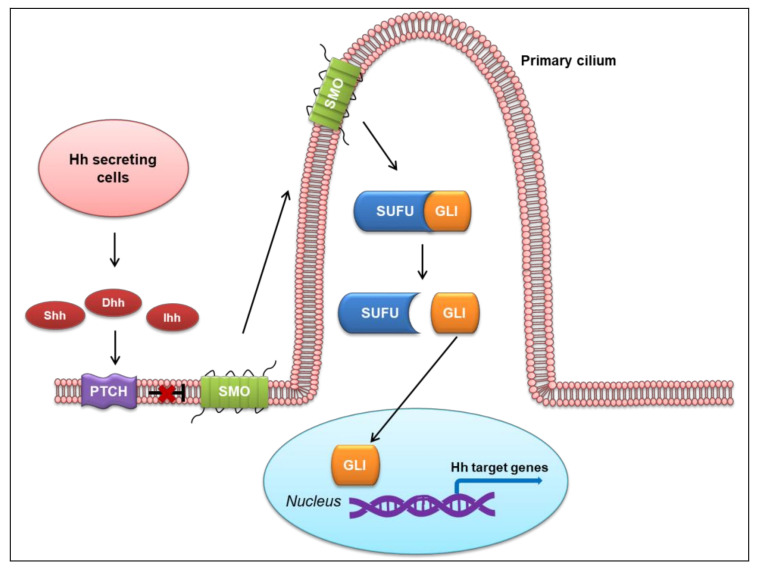
Canonical Hedgehog (Hh) signaling pathway in vertebrates. Shh, sonic hedgehog; Dhh, desert hedgehog; Ihh, Indian hedgehog; PTCH, Patched; SMO, Smoothened; SUFU, Suppressor of fused; GLI, glioma-associated oncogene homolog protein.

Vertebral Hh ligands are secreted from specialized cells, then move across the developing tissue to responsive cells and bind to the surface receptor Patched (PTCH) [22,23]. PTCH is an unconventional receptor, as it does not directly convey the Hh signal to the intracellular components of the pathway. Rather, the binding of an Hh ligand to PTCH alleviates the inhibitory effect of PTCH on a G protein-coupled receptor (GPCR)-like protein, SMO [24,25,26]. In the absence of Hh ligand, PTCH localizes in the membrane and represses SMO activity, preventing its accumulation in the cilium. Upon Hh ligand binding to PTCH, SMO migrates to the tip of the cilium and signals Suppressor of fused (SUFU) to release glioma-associated oncogene homolog proteins (GLIs). GLI then translocates to the nucleus and initiates a signaling cascade through the transcription of Hh target genes. This process overall represents the canonical Hh pathway [12,27,28,29], as illustrated in Figure 1.

### 2.2. Non-Canonical Hh Signaling Pathway

The Hh signaling pathway is also activated through non-canonical mechanisms, which can be SMO-independent GLI activation [30,31,32,33,34] or GLI-independent activation (SMO-dependent or PTCH-dependent mechanism) [35]. In the first type, the signal can circumvent the canonical axis to activate GLI. In esophageal adenocarcinoma, activation of the mTOR/S6K1 pathway induces phosphorylation on serine residue at position 84 in GLI1, whose transcriptional and oncogenic activities are consequently activated [33]. In vitro studies with pancreatic cell lines have shown that ectopic expression of oncogenic KRAS can increase the transcriptional activity of GLI, whereas depletion of oncogenic KRAS significantly downregulates GLI activity [36,37]. In gastric cancer cells, the KRAS-MEK-ERK pathway has a positive regulatory role in GLI expression, but the mechanism remains to be elucidated [38]. Transforming growth factor beta (TGF-β) was found as a potent inducer of both GLI1 and GLI2 expression in various human cell types, including normal fibroblasts and keratinocytes as well as cancer cell lines such as MDA-MB-231 breast carcinoma cells. GLI2 induction by TGF-β is a Smad3-dependent mechanism and mediates subsequent GLI1 activation [39]. The role of the PKC signaling in the activation of the non-canonical Hh pathway is still controversial and may be dependent on the cell type or PKC subtype [40]. In mammalian fetal kidney epithelial cells, PKCδ promotes GLI1 expression, whereas PKCα downregulates GLI1 [41]. Atypical protein kinase C ι/λ (aPKC-ι/λ) has been identified to function downstream of SMO to phosphorylate and activate GLI1 in mouse BCC cell lines [31]. Activation of the PI3K/AKT pathway is essential for GLI activation in the specification of neuronal fates in chicken neural explants [42]. Furthermore, endogenous RAS-MEK and AKT signaling regulate the nuclear localization and transcriptional activity of GLI1 in melanoma [42,43].

Aside from the numerous signals that positively affect GLI activity, there is increasing evidence for non-canonical repression of GLI activity. The tumor repressor p53 suppresses the transcriptional activity of GLI1 by preventing its nuclear localization and reducing the expression. Accordingly, p53 inhibits GLI1-driven neural stem cell self-renewal, tumor growth, and proliferation [44,45]. Numb antagonizes Hh effects on medulloblastoma and cerebellar granule cell progenitors cells by targeting and inhibiting GLI function [46]. Another negative regulatory mechanism of GLI activity applies to the Notch pathway, which displays tumor suppressor function in the skin [47]. Deletion of Notch1 in the skin and primary keratinocytes results in sustained upregulation of GLI2 and development of BCC-like tumors [48].

Alternatively, the Hh signaling pathway can be activated via a GLI-independent but SMO-dependent mechanism. In this mechanism, the activation of the Hh pathway requires the recruitment of small GTPases, such as RhoA, Rac1, or Src. Shh acutely stimulates Rac1 and RhoA via SMO through a G_i_ protein and PI3K-dependent mechanism, which are required for cell migration [49]. Moreover, Shh can signal through the stimulation of Src family kinase in the role of axon guidance [50]. The non-canonical Hh pathway that is activated through a PTCH-dependent mechanism has been found to regulate apoptosis and the cell cycle [51]. In vitro and in ovo studies have suggested that the expression of PTCH in the absence of Hh ligand leads to apoptosis, which can be blocked by the binding of the ligand [52]. Furthermore, a recent study has shown that the Hh pathway modulates cell proliferation via the interaction of phosphorylated cyclin B1 with the large intracellular loop of PTCH between transmembrane domains (TM) 6 and 7 [53]. The binding of Shh ligand to PTCH disrupts the interaction with cyclin B1, allowing its translocation to the nucleus and promoting completion of mitosis [51].

**Figure 2:**
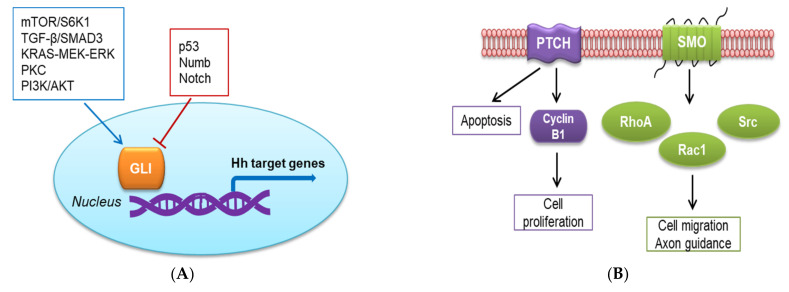
Non-canonical Hedgehog (Hh) signaling pathway. (**A**) SMO-independent GLI activation; (**B**) GLI-independent activation.

The non-canonical pathways involving SMO- or GLI-independent activation are depicted in Figure 2.

## 3. Targeting the Hh Signaling Pathway in Cancer Therapy

### 3.1. Activation of the Hh Signaling Pathway in Cancer

The first link between the Hh signaling pathway and cancer originated from the discovery of PTCH mutations in basal cell nevus syndrome (BCNS, also known as Gorlin syndrome or nevoid BCC syndrome [54,55]), a rare and hereditary form of BCC [56,57]. It is believed that upregulation of the Hh pathway is a unique and major abnormality to drive the development of BCC [10,58]. Furthermore, aberrantly activated Hh signaling has been observed in other types of cancer, such as medulloblastoma, breast cancer, lung carcinoma, and pancreatic cancer [11,13,59,60,61].

In general, three basic models to explain the activation of the Hh signaling pathway in cancer have been proposed [62,63,64]. Type I cancer, the first discovered, harbors Hh pathway-activating mutations and is independent of Hh ligands. This autonomous activation occurs in BCC and medulloblastoma [11,59] and results from loss-of-function mutations in PTCH or SUFU, or gain-of-function mutations in SMO [56,62,65,66]. Particularly, approximately 85% of sporadic BCC has inactivating mutations in PTCH, and 10% has activating mutations in SMO [57,65]. Approximately 30% of medulloblastomas and occasionally rhabdomyosarcomas have abnormal activation of the Hh pathway, which is often due to PTCH or SUFU mutations [11,66,67].

The type II model is autocrine (or juxtacrine)-ligand-dependent. In this model, Hh is both secreted and responded to by the same (or adjacent) tumor cells [59,62]. The overactivation of the Hh signaling pathway in the type II model has been found in various tumors, including stomach, esophageal [68], pancreatic [61], colorectal [69], ovarian and endometrial [70], breast [12,71], prostate [72], lung [73], melanomas [74], gliomas [75], and extracutaneous tumors. The tumor growth in this autocrine manner can be effectively suppressed by Hh neutralizing antibodies or SMO antagonists [14].

The last model, type III cancer, is paracrine-ligand-dependent. In cancer development, Hh ligands are secreted by tumor cells and bind to the PTCH receptor on tumor stromal cells. In a feedback loop, the stromal cells transmit the growth signals to tumor cells, promoting their proliferation and differentiation [59,62,76,77]. The paracrine-ligand-dependent activation appears in colon, pancreas, and prostate cancers [59,62].

The reverse paracrine signaling model has also been proposed in cancer development very recently. In this model, Hh is secreted from the stroma and received by the tumor cells [77]. To date, this mechanism has been identified only in hematological malignancies, such as multiple myeloma, lymphoma, and leukemia [63,78,79]. Stromal cells provide a tumor microenvironment that is favorable for tumor growth, and thus, Hh signaling may also be a potential therapeutic target for the treatment of these hematological cancers [77].

### 3.2. Development of Hh Pathway Inhibitors as Targeted Cancer Therapy

In the light of the aberrant Hh signaling pathway in cancer, enormous efforts have been made to develop inhibitors targeting this pathway. Theoretically, every component of the Hh signaling pathway can serve as a target for cancer treatment. However, targeting SMO receptor displays some advantages over the other Hh pathway members. Inhibiting SMO can be more flexible as it is a membrane protein; various design strategies can be learned from other GPCR inhibitors, as SMO is a member of the GPCR proteins [80]. On the other hand, due to the complex mechanisms of action of GLI members, the library of GLI1 antagonists in clinical trials is not as extensive as that for SMO [81].

To date, two SMO inhibitors, vismodegib and sonidegib, have been approved by the FDA for treating BCC. The first SMO antagonist is a naturally occurring alkaloid called cyclopamine, which is found in the corn lily [82]. Subsequent studies found that it bound to SMO and inhibited the activation of downstream Hh target genes [83]. Using mouse models of Hh-dependent tumorigenesis, the effect of this compound has been widely evaluated in various types of cancer with promising outcomes [61,68,72,73,84,85,86]. However, the poor oral bioavailability, acid sensitivity, and some degrees of specificity of cyclopamine limit its therapeutic usage in clinical study [87,88]. Therefore, an intensive study has been conducted to develop cyclopamine derivatives targeting SMO to inhibit the Hh signaling. In recent years, several SMO inhibitors have been generated and gained success as targeted cancer therapy. Vismodegib (GDC-0449, Erivedge^®^) has a higher potency and more favorable pharmaceutical properties than cyclopamine. Vismodegib is the first-in-class SMO antagonist approved by the FDA in 2012 after successful clinical trials in patients with locally advanced and metastatic BCC [89,90]. The pivotal phase II ERIVANCE trial showed that the objective response rate was 47.6% for locally advanced BCC and 33.3% for metastatic BCC, at 21 months, with a median response and progression-free survival (PFS) duration of 9.5 months [89,90].

Sonidegib (Erismodegib, NVP-LDE-225, LDE-225, Odomzo^®^) was discovered in 2010. This compound is a potent and selective SMO antagonist with high tissue penetration and the ability to cross the blood–brain barrier with good oral bioavailability [91]. After vismodegib, sonidegib became the second SMO antagonist approved in 2015 for patients with locally advanced, recurrent BCC, based on the results of the phase II BOLT trial [92,93]. In this trial, two different once-daily doses (800 or 200 mg) were given to 230 patients with metastatic or locally advanced BCC. Among those with locally advanced BCC, the objective response rates after 30 months were 38% and 43% in the 800 and 200 mg dosage groups, respectively. In the patients with metastatic BCC, the objective response rates were 17% and 15%, respectively [93].

Glasdegib (PF-04449913, Daurismo^®^) is a benzamide derivative with potent and selective activity [94]. In 2018, glasdegib was approved for combination with low-dose cytarabine (LDAC) for the treatment of patients with newly diagnosed acute myeloid leukemia who are 75 years or older, ineligible for intensive chemotherapy. Approval was based on the results of the phase II BRIGHT 1003 trial, which showed that combining LDAC with glasdegib reduced the risk of death by 49%, compared with the rate under LDAC treatment alone [95].

Multiple novel SMO inhibitors including saridegib and taladegib are in active clinical trials. Saridegib (patidegib, IPI-926) is the only semi-synthetic derivative of cyclopamine among SMO inhibitors. Preliminary findings from a phase Ib/II study of IPI-926 in combination with gemcitabine in patients with untreated metastatic pancreatic cancer showed partial response in more than 30% of the patients, with a median PFS of 5.5 months [96]. The drug was well-tolerated with mild side effects [97].

Taladegib (LY2940680) is a phthalazine derivative currently in a phase II trial in combination with chemotherapy and radiotherapy against localized adenocarcinoma of the esophagus or gastroesophageal junction. Notably, taladegib has shown efficacy in cases of SMO-D473H mutation, which causes drug resistance to vismodegib [98].

Itraconazole, an anti-fungal agent, is found to inhibit SMO accumulation in the cilium by binding to a site on the SMO receptor different from that of cyclopamine [99]. Itraconazole efficiently inhibits medulloblastoma and BCC growth in allograft models, showing synergistic effects in combination with cyclopamine [99]. In preclinical models, the combination of itraconazole and arsenic trioxide (ATO) efficiently inhibited the proliferation of Hh-driven medulloblastoma [100]. Currently, itraconazole is under phase II clinical trials for the treatment of BCC [101], metastatic prostate cancer, and non-small cell lung cancer [88].

Several other compounds have been shown to be effective in preclinical models. However, their studies in clinical trials have been limited. BMS-833923 (XL139) has recently been tested in cell lines and mouse xenograft models of cholangiocarcinoma [102,103]. BMS-833923 is also being studied in early phase trials as a single agent in various types of cancer, including multiple myeloma, chronic myeloid leukemia, and small cell lung cancer. However, this agent was withdrawn from the company pipeline in 2014, but the detailed reason was not disclosed [104]. CUR61414 is a member of the aminoproline class that has been identified in the screening for Hh inhibitors. CUR61414 showed moderate inhibition against the Hh pathway via direct binding to SMO and demonstrated significant antitumor efficacy in vivo [105]. However, its clinical translation was suspended in phase I clinical trials due to unsatisfactory results [104]. TAK-441 is a pyridine-4-one derivative that potently inhibits Hh signal transduction in vitro. Oral administration of TAK-441 completely prevented tumor growth in a mouse medulloblastoma allograft model without significant toxicity, and the drug showed an excellent pharmacokinetic profile [106,107]. This compound showed a good inhibitory effect in SMO-D473H mutant cells [107] and was advanced to phase I clinical evaluation in 2010. However, TAK-441 was suspended in 2013 due to project prioritization [108].

Finally, LEQ-506 (NVP-LEQ506) was discovered through a cell-based high-throughput screening of compounds with a phthalazine scaffold [109]. Appealingly, this drug showed efficacy in a cell line carrying SMO-D473H mutation and in a xenografted mouse model, preventing the tumor proliferation. In addition, LEQ-506 showed an optimal pharmacokinetic profile and ability to penetrate the blood–brain barrier [110]. Thus, LEQ-506 has been moved into phase I trials, as a backup for sonidegib [104].

**Figure 3:**
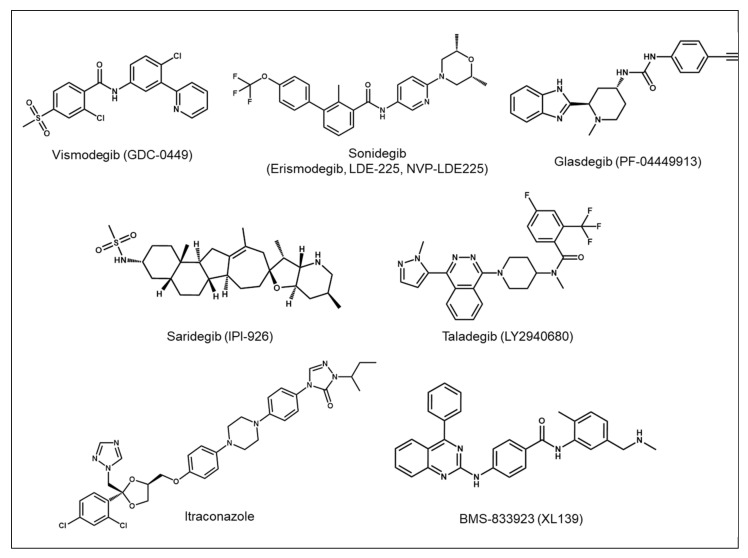
Chemical structures of the selected SMO inhibitors.

The crystal structure of human SMO receptor suggests that SMO antagonists bind to a long and narrow pocket enclosed by TM helices and extracellular loops with an opening to the extracellular environment [111]. Most SMO inhibitors currently in clinical use are thought to bind to the TM helices, with the exception of itraconazole, which does not compete with BODIPY-cyclopamine (a labeled fluorescent cyclopamine derivative) for SMO binding [99,101]. The binding site for most SMO inhibitors, such as cyclopamine, vismodegib, sonidegib, and taladegib, is located at the entrance of the pocket, while the binding site for SANT-1 is located at the deeper part of SMO in the TM domain [80,112,113,114]. Chemical structures of the selective SMO inhibitors approved by the FDA or under clinical trials are depicted in Figure 3. In addition, SMO inhibitors that have been investigated in clinical trials in patients with various types of cancer are summarized in Table 1. SMO inhibitors have transformed the treatment paradigm for BCC, and studies are in progress to expand their use in other types of cancer. Although initially successful, their efficacy in cancer treatment has been diminished by the development of drug resistance. In the following section of this review, we discuss the mechanisms of resistance to SMO inhibitors and the efforts to overcome this obstacle.

## 4. Mechanisms of Resistance to SMO Inhibitor Therapy

### 4.1. Development of Drug Resistance in the Clinical Context

As in other targeted cancer therapies, development of drug resistance is one of the major hurdles in SMO inhibitor therapy. It was reported in a retrospective study in 2012 that the proportion of treated patients who developed drug resistance during therapy was 21%, with a mean tumor recurrence time of 56.4 weeks, as detected in clinical examination [115].

Resistance to SMO inhibitors can be classified into two types—primary resistance (patients who never respond to the SMO inhibitor therapy) and secondary or acquired resistance (patients who initially respond to SMO inhibitors but develop resistance later during the therapy) [15]. Primary resistance to SMO inhibitors has been found in many cases with BCC. A woman in her 70s was diagnosed with infundibulocystic BCC (a rare variant of BCC), and vismodegib therapy was initiated. After a year of treatment, the size and number of her tumors had not decreased yet, and instead, she experienced several side effects. Whole-exome sequencing confirmed the heterozygosity of SUFU mutation enriched in the blood and tumor. In this case, vismodegib was ineffective because the drug targets SMO in the Hh pathway, but SUFU is the downstream molecule of SMO [116]. A similar case with SUFU mutation was seen in a patient with multiple hereditary infundibulocystic BCC. After 9 months of vismodegib therapy, no response was observed, and thus the treatment was discontinued [117]. Another case with primary resistance to vismodegib came from an 82-year-old woman with metastatic BCC. After 2 months of continuous vismodegib treatment, CT scans showed disease progression at all sites, indicating no response to the therapy. The analysis of primary tumor and pre-treated liver metastasis revealed the SMO mutation [118].

Analogously, secondary resistance to SMO inhibitors in cancer treatment may come from SMO mutations. In a phase 2 clinical trial with vismodegib in BCNS, histological analysis of one resistant tumor showed persistent BCC after an initial response, and an SMO mutation was identified. This finding suggests that resistance to vismodegib is more likely to occur in locally advanced or more aggressive BCCs than in non-locally advanced BCC [119]. This could be explained by the fact that somatic SMO mutations are more frequent in sporadic BCCs [120]. Another case of acquired resistance occurred in a BCC patient who reached complete clinical response to vismodegib after 5 months of therapy. However, the lesion reappeared at 11 months on vismodegib. In genetic analysis, no SMO mutations were detected in the earlier stage of treatment, whereas the sample obtained from the recurrence stage showed the D473Y mutation [118]. The first clinical trial to assess the tumor response to sonidegib therapy in patients with advanced BCC resistant to vismodegib treatment was conducted from 2011 to 2013 with nine patients. The study was terminated due to the lack of responsiveness in any of these patients. SMO mutations previously verified to confer tumors with resistance to one of the SMO inhibitors were identified in five patients in this study, suggesting that patients who had developed resistance to an SMO inhibitor therapy may not respond to the other SMO inhibitors [121]. Therefore, understanding the mechanisms involved in the development of drug resistance is critical to establish therapeutic strategies to obtain durable responses to SMO inhibitor therapy.

### 4.2. Mechanisms of Resistance to SMO Inhibitor Therapy

To date, several mechanisms of resistance to SMO antagonists have been discovered, including variant mutations in the components of the Hh pathway, the activation of the non-canonical Hh pathway, and loss of primary cilia. We collectively summarize these mechanisms of drug resistance in the following section.

#### 4.2.1. Genetic Mutations

- Mutations of SMO

**Figure 4:**
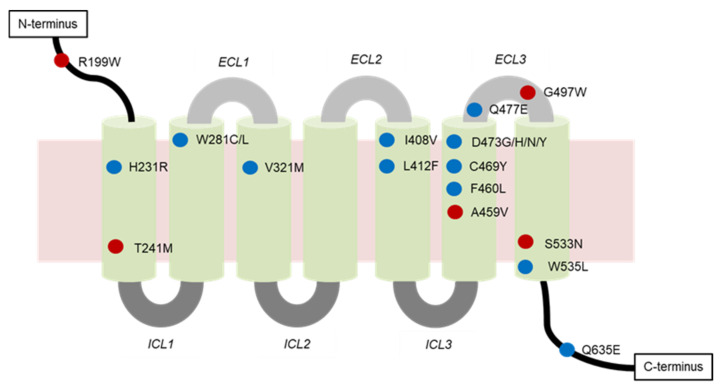
Locations of SMO mutations associated with drug resistance. Amino acid residues in the drug binding pocket are labeled in blue, and the residues in the non-drug binding pocket are in red. ECL, extracellular loop; ICL, intracellular loop.

Genetic analysis of resistant tumors found that SMO mutations, loss of SUFU, and amplification of GLI or Hh target genes can confer resistance to SMO inhibitors. SMO mutations were identified in 50% of resistant BCCs, and in the presence of these mutations, the activation of the Hh signaling pathway was maintained. SMO mutations can be classified into two types—mutations located in the drug binding pocket (DBP) or outside of the DBP (non-DBP) (Figure 4). Spontaneous mutations such as C469, D473, I408, V321, and W281, located in the DBP of SMO, expressed impaired binding of SMO inhibitors [122,123]. Among these mutation sites, D473 is a key residue in SMO for vismodegib binding. D473 could either be directly involved in the binding of vismodegib to the SMO or simply be required to maintain the correct SMO conformation for the binding [124]. Mutations at D473 were identified in 17% of resistant BCCs [123]. This mutation was first discovered through a genetic analysis of a medulloblastoma patient who developed acquired resistance to vismodegib [125]. Binding of vismodegib labeled with ^14^C to human embryonic kidney 293 cells transfected with SMO-wild type (SMO-WT) was seen with high specificity, whereas it showed no specific binding to SMO-D473H. Data from both medulloblastoma cells and allograft mouse models provide additional evidence that the mutation of SMO at this specific aspartic acid residue can confer resistance to vismodegib [125]. Other than D473H, another variation, D473Y, is also found in vismodegib-resistant BCC. In the presence of the D473Y mutation, a considerable conformational change in the binding site is induced, ultimately leading to the total disruption of the stabilizing hydrogen bond network. SMO inhibitor is then shifted away from its optimal position. This mutation translates into an almost two-fold decrease in protein affinity to vismodegib relative to the affinity of the WT receptor [118].

Mutations at W281, V321, I408, and C469 were discovered in a computational docking of vismodegib onto the SMO structure. The SMO-W281C mutant was found to disrupt the interaction between the aromatic indole of the SMO structure at W281 and the pyridine ring of vismodegib by the less bulky sulfur. Furthermore, mutation of Val321 to Met (V321M) is likely to interfere with the positioning of W281, exerting a secondary effect on drug binding. Unlike W281, I408 does not directly contact the drug in this computational model. Instead, it packs against the binding pocket residues H470 and V404 with its delta methyl group. This effect results in changing the conformations of these residues as well as the overall protein backbone, and even affects the binding more strongly. Finally, the substitution of C469 to a bulky tyrosine (C469Y mutation) is predicted to interrupt the conformation of the DBP by its steric effects. These DBP mutations critically impede the functional binding of SMO inhibitors, as they increased the IC_50_ values of vismodegib 12–49-fold over that of SMO-WT [122]. In the structure of the 7-TM SMO receptor, there are seven TM α helices that act in concert to transduce activity, with helices 3, 5, 6, and 7 having pivotal roles in the activation of the receptor. W535L is a mutation previously found on helix 7 and is believed to cause the SMO to be constitutively active (CA) [65]. CA mutants on helix 3 (V321M), helix 5 (L412F), and helix 6 (F460L) complement W535L. These mutations may be hotspots for resistance alleles in Hh-dependent cancers [123].

Surprisingly, SMO mutations at T241, A459, S533, and G497, located distally from the DBP (Figure 4), may also confer drug resistance. This type of mutation destabilizes the SMO architecture to promote activation and decrease its affinity for inhibitors [122,123]. This phenomenon has also been observed for other GPCRs [126]. Both T241M and A459V mutations display increased basal activity over SMO-WT, in line with reduced sensitivity to inhibition by vismodegib. T241M and A459V mutations shifted the IC_50_ values of vismodegib approximately 3- and 9-fold, respectively, in cerebellar granule neuron precursor cells [122]. Another non-DBP mutation, G497W, was also found to be involved in vismodegib resistance. In the presence of the tryptophan mutant residue, the entire region undergoes a conformational rearrangement, thus resulting in a narrowing of the drug entry site. For this reason, SMO inhibitors might be less able to reach the DBP, and hence, less effective in their inhibitory activities. This would eventually allow defining SMO-G497W as a possible biomarker for drug resistance, ultimately enabling the avoidance of unnecessary toxicity effects and cost limitations in cases involving non-responding patients [118]. The SMO mutations associated with drug resistance found in the clinical context are listed in Table 2.

- Mutations of Proteins other than SMO

Focal amplifications of the transcription factor GLI2 and its target gene *CCND1* encoding cyclin D1 are plausible mechanisms of resistance to vismodegib. High cyclin D1 levels likely sustain tumor cell proliferation in the presence of vismodegib, as its expression is no longer reliant on the Hh signaling due to the gene amplification. Similarly, enhanced GLI2 expression by gene amplification could render/maintain the activation of the Hh pathway in tumor cells [124]. Medulloblastoma tumors with alterations in the downstream Hh pathway, such as SUFU or MYCN, may also demonstrate primary resistance to SMO inhibitors [127]. This was confirmed in a study investigating the resistance to sonidegib in in vitro and in vivo xenografts using three different types of medulloblastoma cell lines [128]. Treatment with sonidegib significantly inhibited the proliferation of cells or tumors with a PTCH mutation. However, sonidegib did not affect the proliferation of medulloblastoma with MYCN amplification or SUFU deletion [129].

#### 4.2.2. Activation of the Non-Canonical Hh Pathway

While initial studies identified various mutations in the canonical Hh pathway as the major mechanisms of drug resistance [123], subsequent studies revealed that the resistance could also be driven by the non-canonical Hh signaling [130]. Concurrent activation of AP-1 and TGF-β signaling promotes a non-canonical activation of the Hh signaling pathway, which causes resistance to SMO inhibitors [130]. AP-1 and TGF-β cooperate to induce the transcription of Rho exchange factor and Rho guanine nucleotide exchange factor 17 (Arhgef17), also known as tumor endothelial marker 4. Consequently, Arhgef17 activates RhoA and subsequent actin polymerization, leading to nuclear localization of myocardin-related transcription factor (MRTF). Nuclear MRTF binds to serum response factor (SRF) and acts as a positive transcriptional cofactor for GLI [131]. This sequence leads to the activation of the non-canonical Hh signaling in resistant BCC [130].

Dual-specificity tyrosine-phosphorylation-regulated kinase 1B (DYRK1B) was identified as a critical player in both sensitive and resistant cancers to SMO inhibitors [132]. The DYRK family has been shown to positively or negatively regulate the Hh signaling [133]. As a class I member of the family, DYRK1B has been reported to promote the expression of Hh ligands and suppress the activation of Hh pathway by the autocrine mechanism [134,135]. Interestingly, inhibition of DYRK1B by RNA interference or harmine treatment largely prevented GLI1 expression and moderately reduced GLI2 expression. Based on these findings, small molecule inhibition of DYRK1B was proposed to be a promising approach to target GLI1-dependent cancers resistant to SMO inhibitors [132].

Up-regulation of the insulin-like growth factor 1 receptor-phosphatidylinositol 3-kinase (IGF-1R-PI3K) signaling was discovered as another mechanism of resistance by profiling the differential gene expression pattern in resistant versus sensitive medulloblastoma. Three of the most highly ranked pathways are AKT, phosphatidyl inositol 3,4,5-trisphosphate (PIP3), and IGF-1R, which are directly related to the IGF-1R–PI3K signaling. This finding strongly suggests that the compensatory up-regulation of the IGF-1R-PI3K pathway contributes to the development of resistance. Indeed, the PI3K gene was shown to be up-regulated in 11 of 16 resistant tumors [30].

SRF was also found as a putative cofactor of GLI1 with increased transcriptional activity in resistant BCCs [131]. Active SRF along with its coactivator megakaryoblastic leukemia 1 (MKL1) binds to DNA near Hh target genes and forms an unknown complex with GLI1, causing an amplification of its transcriptional activity [136,137,138]. Nuclear MKL1 is present in the majority of resistant BCCs. In resistant BCCs, both SRF and MKL1 are required for tumor growth and increase the activity of the Hh pathway. Using murine SMO inhibitor-resistant BCC cell lines, knockdown of SRF caused a significant decrease in cell growth and GLI1 mRNA level. Taken together, these findings support the model in which activated SRF and MKL1 maintain the downstream activity of the Hh pathway and are necessary for resistant tumor growth in BCC [131].

Finally, RAS/MAPK activation drives resistance to SMO inhibitors as well as tumor evolution and metastasis in Hh-dependent cancer. Expression of the G12V mutation of pro-oncogene HRAS or HRAS (G12V) and V600E mutations of BRAF induced resistance to SMO inhibitors, such as sonidegib, vismodegib, and LEQ-506, in Shh-subtype medulloblastoma (SMB) cells [139]. Furthermore, MAPK activation is greater in SMB tumors that spontaneously develop resistance to SMO inhibitors, as compared with vehicle-treated, sensitive tumors. Taken together, these data indicate that the activation of RAS/MAPK provides a novel way for cancer cells to evade SMO inhibition [139]. Surprisingly, HRAS (G12V) does not confer resistance by reactivating downstream signaling of SMO. Instead, it enables SMB cells to grow independently on the Hh signaling, and thereby causes resistance to SMO inhibitors [139].

#### 4.2.3. Loss of Primary Cilia

Although the research for mechanisms of drug resistance has deepened our understanding of the activation of the Hh pathway, a new mechanism of resistance has recently been identified just at the surface of cells: loss of primary cilia. This change has been found to confer resistance to sonidegib in medulloblastoma cells [140]. Under normal conditions, cilia harbor the core components of the Hh pathway that are essential for signal transduction [17]. Although the loss of cilia during tumor formation is not completely unexpected, the mechanistic insights of resistance demonstrate an interesting finding that cilia loss protects tumor cells from SMO inhibitors. Using a genome-wide transposon mutagenesis screening in Hh-dependent medulloblastoma cells, SUFU and oral facial digital syndrome 1 (OFD1) were identified as culprit genes [140]. Recurrent mutations in OFD1 result in loss of cilia, and thereby confer resistance to SMO inhibitors. Consequently, resistant tumors lacking cilia ultimately enter a “persister” state of slow and GLI2-dependent growth. In cilia-mutant cells, only the full-length form for GLI2 (GLI2-F) is detected, and its levels are not affected by SMO inhibitors. The surprising experimental evidence indicates that upon losing cilia, the proteolytic processing of GLI2 is impaired, and the truncated repressor form of GLI2 (GLI2-R) is not generated. Consequently, the Hh signaling becomes constitutively active due to the presence of unprocessed GLI2-F. Together, these results suggest that loss of cilia abolishes SMO-dependent full activation of Hh signaling and simultaneously eliminates the truncated repressor form (GLI2-R). Therefore, the cells remain with low but persistent GLI2 activity and Hh pathway transcriptional output. This trade-off enables the cells without cilia to escape drug inhibition and maintain a “persister” state [140].

The results from in vivo studies bolster this finding. OFD1-mutant cells were orthotopically transplanted into mice, and then both the parental and OFD1-mutant cells were observed to undergo tumorigenesis in the brain. Notably, although sonidegib abrogated the growth of the parental cells, the OFD1-mutant cells exhibited complete resistance to sonidegib treatment. Loss of primary cilia was also determined in preclinical in vivo models with acquired resistance. After SMB21 parental cells were transplanted into nude mice, an initial robust response to sonidegib treatment was achieved. However, resistant tumors were spontaneously developed in all the animals. Immunostaining for cilia markers revealed that the resistant tumors showed a higher percentage of unciliated cells than the untreated tumors [140].

**Figure 5:**
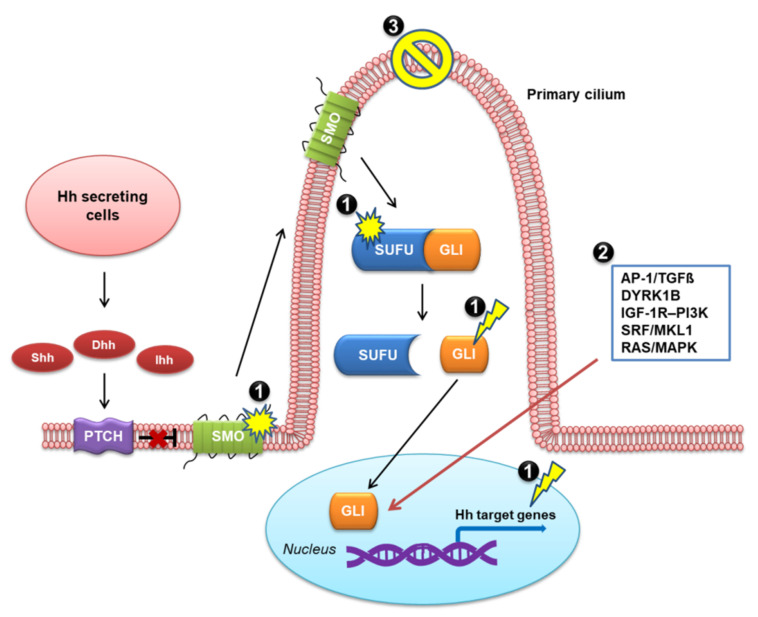
Mechanisms of resistance to SMO inhibitors, including: (1) genetic mutations including SMO mutation, loss of SUFU, and amplification of GLI or Hh target genes; (2) activation of non-canonical Hh pathway; and (3) loss of primary cilia. The black arrows indicate the canonical Hh pathway, and the red arrow indicates the non-canonical Hh pathway.

In the clinical context, analyses of sequencing data from 11 resistant and 48 untreated BCC patients [122] revealed many mutations in ciliary genes. Importantly, mutations in any one of the ciliary genes may lead to the rapid growth of resistant tumors. Together, these results provide both preclinical and clinical evidence that loss of primary cilia constitutes a route for developing resistance to SMO inhibitors [140]. This conclusion opens up a question as to whether reintroducing cilia can be a strategy to reset the Hh signaling and resensitize tumor cells to SMO inhibitors. Although this strategy may offer an advantage against the drug resistance, identifying mechanisms to reintroduce cilia requires an in-depth understanding of cilia assembly and disassembly in normal and cancer cells. Despite these limitations, cilia-regulating signaling pathways in drug-resistant cancers offer new avenues to target ciliary functions for cancer therapy [140]. The possible mechanisms of resistance to SMO inhibitors are summarized below in Figure 5.

## 5. Strategies to Overcome the Resistance to Hh Pathway Inhibitors

### 5.1. Development of Second-Generation SMO Inhibitors

**Figure 6:**
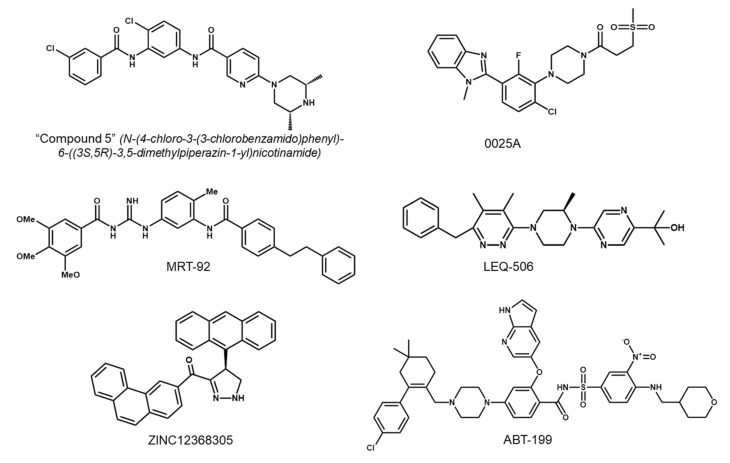
Chemical structures of the selected second-generation SMO inhibitors under investigation.

Since the mechanisms underlying resistance to SMO inhibitors are elucidated, several approaches have been attempted to overcome the resistance in cancer treatment. One approach to overcoming the drug resistance generated by specific mutations in the drug target is to develop second-generation inhibitors retaining anti-cancer activities in the presence of the resistance-conferring mutations. To this goal, a panel of compounds has been screened, particularly focusing on the bis-amide class. The “Compound 5” (N-(4-chloro-3-(3-chlorobenzamido)phenyl)-6-((3S,5R)-3,5-dimethylpiperazin-1-yl) nicotinamide, Figure 6), which was chosen for further investigation from 14 potential candidates, exhibited good pharmacokinetic profiles in mice. This compound has a terminal half-life of approximately 22 h and displays robust activity against both SMO-WT and SMO-D473H mutant [124]. To determine the in vivo efficacy of the “Compound 5”, a vismodegib-resistant allograft model expressing SMO-D477G was used. Although tumor growth in vismodegib-treated animals did not differ from that in the vehicle-treated group, the tumors in animals treated with the “Compound 5” not only stopped growing, but even decreased in size during the relatively short period of treatment. The inhibition of tumor growth was accompanied by downregulation of GLI1 mRNA levels, indicating that the “Compound 5” strongly suppresses Hh signaling in vivo. These findings support the therapeutic potential of the “Compound 5” to circumvent the resistance to conventional SMO inhibitors, which has to be confirmed in clinical trials [124].

In addition, 0025A was discovered as one of the active small molecules targeting SMO from the high-throughput screening platform based on the mechanistic discovery of the Hh signaling pathway [141]. Evidence from in vitro experiments showed that 0025A possessed the ability to bind to both SMO-WT and SMO-D473H mutant. In the competition binding assay, 0025A was able to displace 5 nM BODIPY-cyclopamine from SMO-WT with affinities similar to vismodegib. Moreover, 0025A effectively displaced 5 nM BODIPY-cyclopamine from SMO-D473H, while vismodegib required a high concentration to show only partial effect. The accumulation of SMO in primary cilia and GLI expression upon Hh stimulation were found to be reduced by 0025A. Results from in vivo experiments suggest that 0025A suppresses hair follicle morphogenesis and hair growth in mice. Accordingly, 0025A is a potent antagonist targeting both wild type and mutant SMO receptors in the Hh signaling pathway and may provide a new therapy for refractory cancers [141].

Additional SMO inhibitors, HH-1, HH-13, and HH-20, were also identified from multiple series of benzimidazole derivatives with potent target suppression (IC_50_ < 0.1 μM) in the reporter assays. These inhibitors were able to overcome the acquired drug resistance to first-generation SMO inhibitors by potently targeting SMO-D473H mutation. In GLI1-luciferase reporter assays using 293T cells transfected with SMO-WT and SMO-D473H mutant, vismodegib potently inhibited the activation of SMO-WT, but was poorly active against SMO-D473H mutant. In contrast, both HH-13 and HH-20 retained decent inhibition potency against SMO-D473H, achieving IC_50_ values of less than 0.2 μM. These results identify HH-13 and HH-20 as potent inhibitors capable of targeting naïve and drug-resistant Hh/SMO-driven cancers [142].

Hh003 is also a novel potential SMO inhibitor that has been designed based on tetrahydropyrido (4,3-d)pyrimidine scaffold [143]. Hh003 potently blocked the Hh pathway, suppressing the transcription of Hh target genes such as GLI1 and PTCH1 induced by Hh pathway agonist. Moreover, Hh003 was reported to induce caspase-dependent apoptosis in human colon and pancreatic cancer cells, while vismodegib did not activate an apoptotic response. Mechanistically, Hh003 induced caspase-8 activation, and the silencing of caspase-8 significantly inhibited Hh003-induced apoptosis. Results from in vitro and in vivo studies further confirmed the anti-tumor activity of Hh003. This compound inhibited the growth of various cancer cells, including the human colon cancer HCT116 cell, pancreatic cancer Panc-1 cell, glioblastoma T98G cell, glioblastoma SF295 cell, and gastric cancer AGS cell. Unlike Hh003, vismodegib did not exert cytotoxicity in these cells. In a nude mouse xenograft model of colorectal cancer, Hh003 repressed tumor xenograft, while vismodegib had no effect on tumor growth. Importantly, the mice treated with Hh003 did not show any discernible side effects or body weight loss. The combined Hh inhibitor and apoptosis inducer properties of Hh003 present great potential for the development of novel anti-cancer therapy [144].

A series of acylguanidine and acylthiourea derivatives of SMO inhibitors were synthesized to modulate the Hh signaling pathway in colon cancer. Among these, the compound that belongs to the acylguanidine class emerged as the best lead candidate for a new oral Hh inhibitor. In a GLI-luciferase reporter assay, this compound proved the most active, and its inhibitory activity (IC_50_ = 0.02 µM) was even more potent than that of vismodegib (IC_50_ = 0.05 µM). It also inhibited the growth and viability of tumor cells, such as LS180 human colon carcinoma and HT1080 human fibrosarcoma cells. Moreover, the oral administration of this derivative inhibited the tumor growth of a colon carcinoma xenograft cancer in nude mice [145]. Considering its preferable pharmacokinetic and metabolic profiles, further elucidation of clinical efficacy in cancer patients associated with dysregulation of the Hh pathway may be warranted.

X-ray structures of human SMO receptor bound to several ligands have revealed two types of binding mode of 7-TM-directed antagonists: those binding mostly to extracellular loops (e.g., taladegib) and those binding deeply to the 7-TM cavity (e.g., SANT-1 [146]). Interestingly, MRT-92, another acylguanidine derivative, was predicted to fill the entire 7-TM cavity encompassing the upper extracellular part and the lower cytoplasmic proximal subpocket, and therefore, it shares characteristics of the two types of binding mode. The distinct binding mode of MRT-92 and its potent inhibitory properties against the D473H mutant may provide advantages to overcome the drug resistance caused by SMO mutations [114].

LEQ-506 is found as a potent SMO inhibitor that can overcome the resistance due to SMO-D473H mutation. Unlike sonidegib, LEQ-506 did not induce any difference between the WT and D473H-mutated SMO receptors in their binding mode. In the presence of SMO mutation, sonidegib failed to have an appropriate position in the active site. However, LEQ-506 was in full harmony in the DBP. The mutant residue H473 is not harmful but beneficial to stabilizing the complex between D473H SMO and LEQ-506. While the D473H mutation disrupts the hydrogen bond network of sonidegib with residues R400 and Q477 via conformational change in TM6, this conformational change does not affect the binding of LEQ-506. Detailed insights into the structural and energetic mechanisms of drug resistance will provide an effective strategy to design more promising SMO inhibitors [147].

In another study, the second-generation inhibitor ZINC12368305 showed improved binding affinity to SMO. The results from the docking analysis depict that the binding affinity of ZINC12368305 to SMO-WT is higher than that of vismodegib, with lower docking energy. Furthermore, the calculated docking energies of vismodegib on the binding site of SMO-mutant variants are higher than the docking energies observed for ZINC12368305. Similarly to LEQ-506, the deviation of TM6 in SMO-mutant variants does not affect the binding activity of ZINC12368305 in the DBP [148].

An attractive alternative is using antagonists with a mechanism of action that is clearly distinct from vismodegib. Itraconazole, a systemic antifungal agent that targets cytochrome P450, has recently been shown to inhibit the Hh signaling, although it is significantly less potent than the Hh inhibitors currently in clinical development [99]. Itraconazole purportedly acts on SMO via a mechanism distinct from that of cyclopamine, although exactly how it functions remains to be determined. Considering this, itraconazole may retain its efficacy against vismodegib-resistant SMO mutants.

Finally, ABT-199, a Bcl-2 homology 3 mimetic, is also reported to overcome resistance to SMO inhibitors caused by SMO mutations. ABT-199 was found to suppress the Hh signaling through its function as a competitive inhibitor of oxysterol, probably by targeting the cysteine-rich domain of SMO. ABT-199 suppressed the Smoothened agonist (SAG)-stimulated Hh activity in Light II cells expressing various SMO mutants. Furthermore, in medulloblastoma transgenic mice containing SMO-W539L mutant, ABT-199 treatment at 50 mg/kg twice a day resulted in a remarkable inhibition of tumor growth, whereas vismodegib administered at 25 mg/kg twice a day (the effective dosage in sensitive tumors) exhibited no effect. Collectively, these results show that ABT-199 is also able to overcome resistance to current SMO inhibitors caused by SMO mutations [149]. Chemical structures of the selected second-generation SMO inhibitors under active investigation are shown in Figure 6.

### 5.2. Targeting Downstream Molecules of SMO

Another strategy worth pursuing is the inhibition of the Hh pathway by targeting downstream signals of SMO. GLI antagonists acting on the transcription factor GLI could be an effective strategy against tumors resistant to SMO inhibitors. In a study using mouse embryonic fibroblasts with SMO mutant variants, the efficacy of SMO inhibitors was compared with that of GLI antagonists. As expected, all variants showed partial or complete resistance to vismodegib. However, both GLI2 antagonist ATO [100] and the myristoylated peptide inhibitor of aPKC-ι/λ/GLI [31] were effective in suppressing the activation of the Hh pathway in the presence of any SMO variants, suggesting that GLI antagonists may be useful against SMO inhibitor-resistant tumors. To date, a number of small molecules targeting GLI have been discovered and investigated [81]. GANT58 and GANT61 are GLI inhibitors that interfere with the DNA binding of GLIs [150]. These inhibitors show promising efficacy in blocking tumor cell proliferation both in vitro and in vivo [150,151,152]. ATO is an FDA-approved drug used for the treatment of acute promyelocytic leukemia [153]. This drug has been found to directly interact with GLIs and consequently inhibit the expression of Hh target genes [154]. The combination of ATO and itraconazole may be a therapeutic consideration since three out of five patients with refractory metastatic BCC reached a stable state, despite multiple adverse events [155]. Pirfenidone, an anti-fibrotic drug [156], has been found to selectively destabilize GLI2, thereby suppressing the Hh pathway [157]. Imiquimod, an agonist of the toll-like receptors 7 and 8, directly inhibits Hh signaling through stimulation of GLI phosphorylation mediated by adenosine receptor/PKA [158].

Glabrescione B (GlaB), an isoflavone naturally found in the seeds of *Derris glabrescens*, showed therapeutic efficacy in preclinical models of Hh-dependent medulloblastoma. To overcome its poor water solubility, GlaB was formulated with a self-assembling amphiphilic polymer forming micelles, called mPEG5kDa-cholane. mPEG5kDa-cholane/GlaB possesses favorable pharmacokinetics and negligible toxicity. Remarkably, GlaB loaded in mPEG5kDa-cholane micelles crosses the blood–brain barrier and inhibits tumor growth in a Hh-dependent medulloblastoma orthotopic model. The mPEG5kDa-cholane/GlaB micellar system can be properly exploited in the treatment of patients with medulloblastoma, particularly for those tumors showing resistance to SMO inhibitors or harboring GLI1 hyperactivation by SMO-independent mechanisms [159].

To identify new GLI1 modulators, a pharmacophore-based virtual screening approach was applied. Among 41 chemical entries, three different chemical scaffolds were identified to reduce the transcriptional activity of the Hh pathway. These include SST0673 (α-mangostin), SST0682 (a thiophene derivative), and SST0704 (a pyrazolo(1,5-a)pyrimidine analogue), respectively. These compounds showed no cytotoxicity in non-neoplastic mammary epithelial cells and could be potential candidates as GLI inhibitors for cancer treatment [160]. Further studies are in progress to assure the efficacy of these GLI antagonists for the treatment of various types of cancer resistant to SMO inhibitors.

In addition to these GLI antagonists, casein kinase 1α (CK1α) may be a powerful and innovative approach in treating patients with medulloblastoma resistant to SMO inhibitors. CK1α phosphorylates and destabilizes GLI transcription factors, thereby functioning as a negative regulator of the Hh signaling in mammals [64,161]. A novel brain barrier-permeable CK1α agonist, SSTC3, was tested against *TRP53*-mutant, *MYCN*-amplified medulloblastoma (which was resistant to SMO inhibitors). SSTC3 accumulated in the brain was shown to inhibit the tumor growth of medulloblastoma and suppress metastases in a vismodegib-resistant mouse model. Thus, CK1α activators could address a significant unmet clinical need for patients with medulloblastoma resistant to SMO inhibitors [162].

Histone deacetylases (HDACs) have recently been discovered to be involved in Hh modulation. In particular, HDAC1 has been demonstrated to deacetylate the transcription factors GLI1 and GLI2, whose transcriptional activities are consequently induced [163]. Class I HDAC inhibitors have been found efficacious in suppressing the growth of diverse SMO inhibitor-resistant clones of SMB21 cells. Moreover, a novel HDAC inhibitor, quisinostat, is well-tolerated in mice and robustly inhibits the growth of SMB cells in vitro as well as in vivo. Therefore, targeting HDAC may be therapeutically useful for patients with Shh-dependent medulloblastoma, including the cancer type resistant to SMO inhibitors [164].

### 5.3. Targeting the Non-Canonical Hh Pathway

As an alternative approach to circumvent the resistance to SMO inhibitors, blockade of the activity of non-canonical pathways may be challenged. An in vivo study tested the effect of the PI3K inhibitor GDC-0941 in a medulloblastoma model [165]. Daily administration of GDC-0941 at the dosage of 150 mg/kg significantly reduced the tumor growth in both sensitive and resistant models, indicating that these tumors are dependent on the non-canonical PI3K signaling to maintain GLI activation. Consequently, pharmacological inhibition of PI3K/AKT signaling may also represent a promising therapeutic approach to treat SMO inhibitor-resistant medulloblastoma [124].

Targeting DYRK1B is also suggested as a novel alternative strategy to overcome the drug resistance in GLI1-dependent cancer. A DYRK1 inhibitor (DYRKi) was identified to show an IC_50_ value of 3.7 μM to inhibit reporter Hh gene activity. Daily administration of 100 mg/kg DYRKi demonstrated suitable pharmacokinetic profiles [132]. In addition to DYRKi, the MKL1 inhibitor CCG-1423 was reported to inhibit the tumor growth and GLI1 expression in SMO inhibitor-resistant cell lines. Moreover, systemic MKL1 inhibition by CCG-203971 caused a dramatic decline in tumor growth in allograft models of resistant BCCs. Interestingly, while sensitive BCCs and cell lines potently responded to SMO inhibitors, their responsiveness to CCG-203971 was only weak. These results suggest that refractory tumors are more dependent on MKL1 to grow than the tumors sensitive to SMO inhibitors, allowing MKL1 inhibitors to be more effective than SMO inhibitors in resistant BCCs [131].

Preclinical studies in models of Hh-dependent tumors have revealed the importance of PI3K signaling in the development of resistance to SMO inhibitors [30]. Subsequently, a clinical trial to evaluate the anti-cancer effect of combined Hh and PI3K inhibition in BCC patients was conducted using sonidegib and buparlisib, a pan-PI3K inhibitor. Due to early termination of the study resulting from toxicity, the sample size for overall response rate determination was limited to seven patients. The overall response rate was 14.3%. Unfortunately, overall median PFS was 13.8 months, which was comparable to the long-term effect of a single agent, vismodegib or sonidegib, in SMO inhibitor-naïve patients with advanced BCCs [90,166]. Future studies with better tolerated PI3K inhibitors [167] are still warranted, particularly as a potential salvage therapy for SMO inhibitor-resistant advanced BCCs [168].

### 5.4. Genetic Prescreening before Initiating Cancer Therapy with Hh Inhibitors

As described above, a patient with infundibulocystic BCC was reported to show primary resistance to vismodegib therapy [116]. Based on the sequencing results, this resistance was attributed to the mutation in SUFU, the downstream component of SMO in the Hh pathway. This case emphasizes the importance of genetic prescreening before initiating therapy. Prescreening with genetic analysis would have excluded vismodegib as one of the therapeutic options in this patient, and the patient would have been saved from unnecessary side effects [116]. Thus, genetic analysis before initiating SMO inhibitor therapy may provide an opportunity to evade primary resistance, if any, which can help to establish the optimal customized therapy.

## 6. Conclusions

Uncontrolled activation of the Hh signaling pathway is known to be associated with various types of cancer, including BCC, medulloblastoma, pancreatic cancer, breast cancer, and small cell lung carcinoma. Therefore, the Hh pathway has gained intensive attention as an enticing target for cancer treatment. Enormous efforts have been made to develop specific inhibitors targeting molecular components of this pathway. Consequently, various small molecule SMO inhibitors, such as vismodegib and sonidegib, have been successfully approved for BCC therapy. Unfortunately, however, their reputation has been tarnished by the emergence of resistance to these drugs during therapy. To circumvent the resistance, it is crucial to understand the mechanisms underlying the primary as well as acquired resistance of tumors to conventional SMO inhibitor therapy. In this review, we briefly overviewed canonical and non-canonical Hh pathways and their roles in cancer. In addition, we summarized SMO inhibitors in clinical trials as targeted cancer therapy. We then addressed the mechanisms underlying the resistance of tumors to SMO inhibitors and the main strategies that could be applied to overcome this drug resistance.

Resistance of tumors to SMO inhibitor therapy can be explained by at least three mechanisms—genetic mutations of SMO and other signaling molecules in the canonical Hh pathway, activation of the non-canonical Hh pathway, and loss of primary cilia. Firstly, point mutations in the components of the Hh pathway in resistant tumors have recently been uncovered at the level of SMO and its downstream molecules. Alternatively, activation of the non-canonical Hh pathway, which bypasses the canonical pathway to maintain GLI activity, drives tumor growth and enhances metastatic behavior in resistant tumors. Finally, a distinct mechanism is also presented to confer resistance of tumors to SMO inhibitors via loss of primary cilia. When tumor cells lose their primary cilia, a ”persister” state with low output of the Hh signaling is maintained, allowing the tumor cells to evade inhibition by SMO antagonists.

Several strategies have been proposed to overcome the drug resistance in cancer treatment. The first approach is to develop novel and potent second-generation SMO inhibitors that retain their anti-cancer activities even in the presence of the resistance-conferring mutations. In addition, targeting downstream components of SMO in the Hh pathway or signaling molecules involved in the non-canonical pathway can be a promising alternative challenge to overcome the resistance to SMO inhibitors. Moreover, genetic prescreening may provide an opportunity to avoid primary resistance to SMO inhibitors. Although these strategies may offer advantages against the drug resistance, further studies are necessary to avoid emerging resistance to Hh inhibitors and establish optimal customized regimens with improved therapeutic efficacy to treat various types of cancer including BCC.

## Figures and Tables

**Table 1:** SMO inhibitors under clinical trials in patients with various types of cancer. Data from https://clinicaltrials.gov (accessed on 30 August 2021).

SMO Inhibitor	Phase	Cancer Type	Status	ClinicalTrials.gov Identifier #
BMS-833923 (XL139)	Phase 1 and 2	Chronic myeloid leukemia	Completed	NCT01218477
	Phase 2	Leukemia	Terminated	NCT01357655
Glasdegib (PF-04449913)	Phase 2	Acute myeloid leukemia	Completed	NCT01546038
	Phase 2	Acute myeloid leukemia	Completed	NCT01841333
	Phase 2	Myelodysplastic syndrome Chronic myelomonocytic leukemia	Completed	NCT01842646
	Phase 2	Myelofibrosis	Terminated	NCT02226172
	Phase 1 and 2	Acute myeloid leukemia	Recruiting	NCT03390296
	Phase 3	Acute myeloid leukemia	Ongoing	NCT03416179
	Phase 1 and 2	Glioblastoma	Recruiting	NCT03466450
	Phase 2	Acute myeloid leukemia	Ongoing	NCT04051996
	Phase 3	Acute myeloid leukemia Myelodysplastic syndrome Chronic myelomonocytic leukemia	Ongoing	NCT04842604
Itraconazole	Phase 2	Basal cell carcinoma	Completed	NCT01108094
Saridegib (patidegib, IPI-926)	Phase 1 and 2	Pancreatic cancer	Completed	NCT01130142
	Phase 2	Chondrosarcoma	Completed	NCT01310816
	Phase 2	Myelofibrosis	Completed	NCT01371617
	Phase 2	Basal cell nevus syndrome	Completed	NCT02762084
	Phase 2	Basal cell carcinoma	Completed	NCT02828111
	Phase 3	Basal cell nevus syndrome	Completed	NCT03703310
	Phase 2	Basal cell carcinoma	Terminated	NCT04155190
	Phase 3	Basal cell nevus syndrome	Ongoing	NCT04308395
Sonidegib (Erismodegib, LDE-225, NVP-LDE-225)	Phase 2	Basal cell nevus syndrome	Completed	NCT00961896
	Phase 1 and 2	Medulloblastoma	Completed	NCT01125800
	Phase 2	Basal cell carcinoma	Completed	NCT01327053
	Phase 2	Basal cell nevus syndrome	Completed	NCT01350115
	Phase 2	Medulloblastoma	Completed	NCT01708174
	Phase 2	Breast cancer	Withdrawn	NCT01757327
	Phase 1 and 2	Myelofibrosis	Completed	NCT01787552
	Phase 2	Multiple myeloma	Ongoing	NCT02086552
	Phase 2	Multiple myeloma	Terminated	NCT02254551
	Phase 2	Basal cell carcinoma	Terminated	NCT02303041
	Phase 2 and 3	Basal cell carcinoma	Withdrawn	NCT03070691
	Phase 2	Basal cell carcinoma	Recruiting	NCT03534947
	Phase 2	Medulloblastoma	Not yet recruiting	NCT04402073
Taladegib (LY2940680)	Phase 1 and 2	Small cell lung cancer	Terminated	NCT01722292
	Phase 1 and 2	Esophageal junction cancer	Ongoing	NCT02530437
	Phase 2	Idiopathic pulmonary fibrosis	Recruiting	NCT04968574
Vismodegib (GDC-0449)	Phase 2	Colorectal cancer	Completed	NCT00636610
	Phase 2	Ovarian cancer	Completed	NCT00739661
	Phase 2	Basal cell carcinoma	Completed	NCT00833417
	Phase 2	Small cell lung carcinoma	Completed	NCT00887159
	Phase 2	Medulloblastoma (adult)	Completed	NCT00939484
	Phase 2	Basal cell nevus syndrome	Completed	NCT00957229
	Phase 2	Ovarian cancer Basal cell carcinoma Colorectal cancer	Completed	NCT00959647
	Phase 1 and 2	Pancreatic cancer	Completed	NCT01064622
	Phase 2	Pancreatic cancer	Completed	NCT01088815
	Phase 2	Pancreatic cancer	Completed	NCT01195415
	Phase 2	Basal cell carcinoma	Completed	NCT01201915
	Phase 2	Medulloblastoma (pediatric)	Completed	NCT01239316
	Phase 2	Chondrosarcoma	Ongoing	NCT01267955
	Phase 2	Basal cell carcinoma	Completed	NCT01367665
	Phase 2	Basal cell carcinoma	Completed	NCT01700049
	Phase 2	Basal cell carcinoma	Completed	NCT01815840
	Phase 2	Acute myelogenous leukemia Myelodysplastic syndrome	Terminated	NCT01880437
	Phase 2	Basal cell carcinoma	Terminated	NCT01898598
	Phase 2	B-cell lymphoma Chronic lymphocytic leukemia	Terminated	NCT01944943
	Phase 2	Solid tumors	Ongoing	NCT02091141
	Phase 4	Basal cell carcinoma	Ongoing	NCT02436408

**Table 2:** SMO mutations associated with drug resistance found in the clinical context.

SMO Mutation	Location of Mutation	SMO Inhibitor	Type of Resistance	Cancer Type
A459V	Non-DBP ^1^	Vismodegib	Secondary	BCC ^2^
C469Y	DBP	Vismodegib	Secondary	BCC
D473G	DBP	N.D. ^3^	Secondary	BCC
D473H		Vismodegib	Secondary	BCC, MB ^4^
D473H		Sonidegib	N.D.	N.D.
D473N		N.D.	Primary	N.D.
D473Y		Vismodegib and sonidegib	Secondary	BCC
E518A	N.D.	Vismodegib	N.D.	N.D.
F460L	DBP	Vismodegib	N.D.	BCC
G497W	Non-DBP	Vismodegib	Primary	BCC
H231R	DBP	Vismodegib	Secondary	BCC
H304Y	N.D.	N.D.	N.D.	BCC
I408V	DBP	Vismodegib	Secondary	BCC
L225R	N.D.	Sonidegib	N.D.	MB
L412F	DBP	N.D.	Primary	BCC, meningiomas, ameloblastoma
N476K	N.D.	N.D.	N.D.	BCC
Q476	DBP	N.D.	N.D.	BCC
Q477E	DBP	Vismodegib	Secondary	BCC, MB
Q581R	N.D.	N.D.	N.D.	BCC
Q635E	DBP	N.D.	N.D.	N.D.
R168H	N.D.	N.D.	N.D.	BCC
R199W	Non-DBP	N.D.	Primary	BCC
R302K	N.D.	N.D.	N.D.	BCC
S533N	Non-DBP	N.D.	Primary	BCC, primitive neuroectodermal tumors
T241M	Non-DBP	Vismodegib	Primary	BCC
V321M	DBP	Vismodegib	Secondary	BCC
W281C	DBP	Vismodegib	Secondary	BCC
W281L		N.D.	Secondary	BCC
W535L	DBP	N.D.	Primary	BCC, meningiomas
W549X	N.D.	N.D.	N.D.	BCC

^1^ DBP, drug binding pocket; ^2^ BCC, basal cell carcinoma; ^3^ N.D., not defined; ^4^ MB, medulloblastoma.

## Data Availability

Not applicable.
